# Early diagnosis of ovarian cancer based on methylation profiles in peripheral blood cell-free DNA: a systematic review

**DOI:** 10.1186/s13148-023-01440-w

**Published:** 2023-02-14

**Authors:** Simone Karlsson Terp, Malene Pontoppidan Stoico, Karen Dybkær, Inge Søkilde Pedersen

**Affiliations:** 1 Department of Molecular Diagnostics, Aalborg University Hospital, 9000 Aalborg, Denmark; 2 Department of Clinical Medicine, Aalborg University, 9000 Aalborg, Denmark; 3 Department of Hematology, Aalborg University Hospital, 9000 Aalborg, Denmark; 4 Clinical Cancer Research Center, Aalborg University Hospital, 9000 Aalborg, Denmark

**Keywords:** Ovarian cancer, Biomarker, Diagnosis, Systematic review, Epigenetics, DNA methylation, Cell-free DNA, Circulating tumor DNA, Liquid biopsy

## Abstract

Patients diagnosed with epithelial ovarian cancer (OC) have a 5-year survival rate of 49%. For early-stage disease, the 5-year survival rate is above 90%. However, advanced-stage disease accounts for most cases as patients with early stages often are asymptomatic or present with unspecific symptoms, highlighting the need for diagnostic tools for early diagnosis. Liquid biopsy is a minimal invasive blood-based approach that utilizes circulating tumor DNA (ctDNA) shed from tumor cells for real-time detection of tumor genetics and epigenetics. Increased DNA methylation of promoter regions is an early event during tumorigenesis, and the methylation can be detected in ctDNA, accentuating the promise of methylated ctDNA as a biomarker for OC diagnosis. Many studies have investigated multiple methylation biomarkers in ctDNA from plasma or serum for discriminating OC patients from patients with benign diseases of the ovaries and/or healthy females. This systematic review summarizes and evaluates the performance of the currently investigated DNA methylation biomarkers in blood-derived ctDNA for early diagnosis of OC. PubMed’s MEDLINE and Elsevier’s Embase were systematically searched, and essential results such as methylation frequency of OC cases and controls, performance measures, as well as preanalytical factors were extracted. Overall, 29 studies met the inclusion criteria for this systematic review. The most common method used for methylation analysis was methylation-specific PCR, with half of the studies using plasma and the other half using serum. *RASSF1A*, *BRCA1*, and *OPCML* were the most investigated gene-specific methylation biomarkers, with *OPCML* having the best performance measures. Generally, methylation panels performed better than single gene-specific methylation biomarkers, with one methylation panel of 103,456 distinct regions and 1,116,720 CpGs having better performance in both training and validation cohorts. However, the evidence is still limited, and the promising methylation panels, as well as gene-specific methylation biomarkers highlighted in this review, need validation in large, prospective cohorts with early-stage asymptomatic OC patients to assess the true diagnostic value in a clinical setting.

**Supplementary Information:**

The online version contains supplementary material available at 10.1186/s13148-023-01440-w.

## Background

Epithelial ovarian cancer (OC) is the most lethal gynecological malignancy, with a 5-year survival rate of 49% [1, 2]. Most patients are diagnosed at an advanced stage as early stages often are asymptomatic or with unspecific symptoms [2]. When OC is diagnosed at an early stage, the 5-year survival rate is above 90% [1, 2], highlighting the need for tools for early diagnosis.

In search for improvement of survival rates, large clinical screening trials have been conducted combining serum Cancer Antigen 125 (CA-125) and transvaginal ultrasound [3, 4]. These trials, as well as years of extensive research and development of risk score algorithms for early diagnosis of OC, have been unsuccessful in improving survival rates [5, 6].

Liquid biopsy is a minimal invasive blood-based approach that has gained increasing interest in the past decade as a potential screening tool using cancer-specific biomarkers from cell-free DNA (cfDNA) [7]. cfDNA is DNA shed by normal cells into the blood circulation as well as by tumor cells, then termed circulating tumor DNA (ctDNA). ctDNA provides an opportunity for real-time detection of tumor genetics and epigenetics as ctDNA contains most of the genetic and epigenetic information of the tumor, irrespective of sub-clonal distribution [8, 9].

Increased DNA methylation of promoter regions is an early event during tumorigenesis that alters the expression of tumor-suppressor genes [8, 10, 11]. Methylation of cytosines occurs in cytosine–phosphate–guanine (CpG)-rich regions (CpG islands) and is a relatively stable modification of the DNA [8, 10, 11]. In addition, DNA methylation is cancer- and tissue-specific and can be detected in ctDNA and therefore serves as a promising biomarker for early detection of OC [8, 10–12].

No single gene-specific methylation biomarker or panel of methylation biomarkers has yet been implemented for early diagnosis of OC. However, many studies have investigated the potential of multiple DNA methylation biomarkers in cfDNA from plasma or serum to discover gene-specific methylation biomarkers that can differentiate OC patients from patients with benign diseases of the ovaries and/or healthy controls.

When evaluating the clinical utility of a biomarker for diagnosis or population screening, high specificity is required as a low false-positive rate can result in many healthy people being subjected to potential harm from unnecessary procedures or treatment [13, 14]. In addition, when screening for a severe disease, high sensitivity is required as patients otherwise can go undiagnosed with potentially detrimental consequences to follow [14].

The Early Detection Research Network (EDRN) has provided guidelines for evaluating the performance of potential biomarkers, although no set threshold for performance has been suggested as a gold standard [14]. EDRN has provided an example of a hypothetical biomarker for ovarian cancer screening, which detected 80% of OC patients, and calculated that the biomarker had to have a corresponding specificity of no less than 92.5%, whereas a biomarker with a sensitivity of 100% required a specificity above 90.6% [14].

In this systematic review, we will summarize and evaluate the performance of the current DNA methylation biomarkers in blood-derived cfDNA for early diagnosis of OC.

## Methods

This systematic literature review was conducted following the Preferred Reported Items for Systematic Reviews and Meta-Analysis (PRISMA) guidelines [15].

### Literature search

PubMed’s MEDLINE and Elsevier’s Embase were systematically searched for eligible articles. The search string included three main topics, each containing multiple search terms representing the search topic. The search topics were (1) early diagnosis, (2) ovarian cancer, and (3) methylation biomarker/cfDNA/ctDNA. The full search string used in both databases is presented in Additional file 1. The literature search was conducted on April 4, 2022 and was performed with no limitation on the date of publication. The search in Embase excluded conference abstracts, editorials, letters, notes, and short surveys.

The initial screen of title and abstract, as well as full-text assessment of eligibility, was conducted by two authors (SKT and MPS). SKT and MPS performed the literature screen and assessment independently and blinded to each other using Covidence systematic review software [16]. Additional studies were identified by assessment of the bibliographies of included studies and relevant reviews related to the topics of this review.

### Eligibility criteria

Studies were included in the review if fulfilling the following inclusion criteria: (1) evaluating methylation biomarkers, (2) focusing on ovarian cancer, (3) including plasma or serum samples for analyzing cfDNA/ctDNA in methylation analysis, with no limitation on assay type, (4) original research articles, with no limitation on date of publication, and 5) published in English. Studies were excluded if they only included in silico analysis.

Any discrepancies regarding article suitability were solved by consulting two other authors (ISP and KD).

### Data extraction

Two authors (SKT and MPS) critically reviewed included articles and extracted data manually into an Excel spreadsheet. Data concerning cohort size, patient characteristics, specimens, candidate biomarker genes, preanalytical factors, analytical methods, and performance measurements were extracted. The quality of the included articles was assessed using the Newcastle–Ottawa scale [17].

### Statistical analysis

The Clopper–Pearson exact 95% confidence interval was calculated for sensitivity and specificity when data were available using R version 4.2.1 (R Project for Statistical Computing).

## Results

### Literature overview

**Fig. 1:**
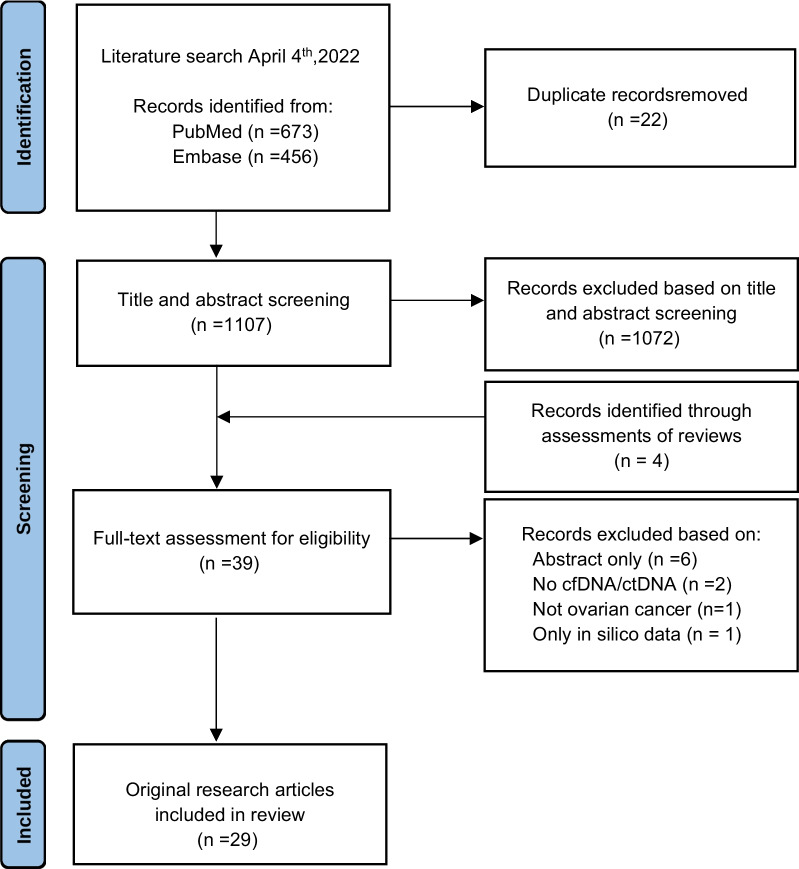
A PRISMA flowchart displaying the identification, screening, and inclusion process of the systematic review. The literature search was conducted on April 4, 2022

The study selection process is illustrated in Fig. 1 by a PRISMA flow chart. Records were identified through searches in PubMed and Embase, with 673 records identified in PubMed and 456 records identified in Embase. Screening for duplicates identified 22 duplicate records that were removed, leaving 1107 records for screening of title and abstract.

From screening titles and abstracts as well as reviewing reviews with relevant topics, 39 records were included for full-text assessment. In the full-text assessment, six records were excluded as they only provided abstracts and no original research paper. Two records were excluded as they did not analyze cfDNA or ctDNA, and one record was excluded as it did not concern ovarian cancer. In addition, one record was excluded as it only analyzed in silico data. After full-text assessment, 29 articles were included in the final analysis.

The quality of the included studies was assessed to be good for six studies, moderate for 18 studies, moderate-to-poor for three studies, and poor for two studies (Additional file 2).

### Study characteristics

Table 1 summarizes all studies that investigated methylation patterns of specific genes, regions, or panels which were published between 2004 and 2022.

**Table 1:** Characteristics of the included studies

Author (et al.)	Year	OC (no.)	Controls (no.)	Histological subtype	Stage	Initial target gene(s) selection and clinical purpose of biomarker
de Caceres [18]	2004	50 (T)	10^b^/20^h^ (T)	Papillary serous Endometrioid Mucinous Clear cell Transitional cell	I/III/IV	Previous research Detection
Su [19]	2009	26 (T)	20^b^ (T)	Serous Endometrial Mucinous	N/A	Previous research Detection/prognostic
Melnikov [20]	2009	33 (T)	33^h^ (T)	Papillary serous	III/IV	Global screening Detection
BonDurant [21]	2011	21 (T)	7^h^ (T)	Serous	I–IV	Global screening Detection
Campan [22]	2011	16 (T)	8^h^ (T)	Serous Endometrioid Mucinous	III/IV	Global screening Detection
Häfner [23]	2011	32 (T)	30^b^/20^h^ (T)	Serous Endometrioid Papillary Clear cell Neuroendocrine	II–IV	Previous research Detection
Liggett [24]	2011	30 (T)	30^b^/30^h^ (T)	Serous	III/IV	Semi-global screening Detection
Dong [25]	2012	36 (T)	25^h^ (T)	Serous Endometrioid Mucinous	I–IV	Previous research Detection
Wang [26]	2013	60 (T)	30^b^/30^h^ (T)	Serous Endometrioid Mucinous Clear cell	I–III	Previous research Detection
Zhang [27]	2013	87 (T) 39 (V)	53^b^/62^h^ (T) 29^b^ (V)	Serous Endometrioid Mucinous Clear cell Mixed	I–IV (T) I–III (V)	Previous research Detection
Wu [28]	2014	47 (T)	14^b^/10^h^ (T)	Serous Endometrioid Mucinous	I–IV	Previous research Detection
Zhou [29]	2014	45 (T)	60^h^ (T)	Serous Endometrioid Mucinous Clear cell Undifferentiated	I–IV	Previous research Detection
Wang [30]	2015	71 (T)	43^b^/80^h^ (T)	Serous Endometrioid Mucinous Clear cell	I–IV	Previous research Detection
Giannopoulou [31]	2017	59 (T)	51^h^ (T)	High grade serous	N/A	Previous research Prognostic
Swellam [32]	2017	90 (T)	50^b^/30^h^ (T)	Serous Endometroid Mucinous	I–IV	Previous research Detection
Wang [33]	2017	71 (T)	43^b^/80^h^ (T)	N/A	I–IV	Previous research Detection
Widschwendter [34]	2017	29 (T) 48 (V)	119^b^/21^h^ (T) 154^b^/41^h^ (V)	High grade serous Endometrioid Clear cell Mucinous Carcinosarcoma	I–IV	Global screening Detection
Giannopoulou [35]	2018	50 (T)	51^h^ (T)	Serous	I–IV	Previous research Prognostic
Dvorská [36]	2019	33 (T)	5^b^/3^bc−oc^/9^h^ (T)	Serous Serous papillary Endometrioid Mucinous Clear cell	N/A	Previous research Detection/prediction of risk
Kumar [37]	2019	53 (T)	12^b^/7^lmp^/15^h^ (T)	Serous Endometroid Mucinous Clear cell	I–IV	Previous research Detection/prognostic
Liu [11]	2020	27 (T) 12 (V)	1521^h^ (T) 610^h^ (V)	Fallopian tube Primary peritoneal	N/A	Global screening Detection
Miller [38]	2020	26 (T) 8 (V)	41^h^ (T) 12^h^ (V)	Serous Non-serous	N/A	Previous research Detection
Singh [39]	2020	45 (T)	25^h^ (T)	Serous Mucinous	I–IV	Previous research Detection
Faaborg [40]	2021	26 (V) 79 (A)	64^h^ (T)	Low-grade serous High-grade serous Endometrioid Mucinous	I–IV	Previous research Detection
Miller [41]	2021	38 (T)	20^h^ (T)	N/A	III/IV	Previous research Detection
Singh [42]	2021	45 (T)	25^h^ (T)	Serous Mucinous	I–IV	Previous research Detection
Tserpeli [43]	2021	84 (T) 49 (T)	27^h^ (T)	High grade serous	III/IV	Previous research Prognostic
Marinelli [44]	2022	91 (T)	91^h^ (T)	Low grade serous High grade serous Endometrioid Mucinous Clear cell	I–IV	Global screening Detection
Tomeva [45]	2022	19 (T)	8^h^ (T)	N/A	II/III	Previous research Detection

*A* analysis cohort, *BC-OC* ovarian cancers subsequent to breast cancer, *b* benign, *h* healthy, *LMP* low malignant potential, *N/A* not available, *OC* ovarian cancer, *T* training/test cohort, *V* validation cohort

Generally, the included case numbers were rather low, ranging from 16 to 91 for training cohorts and eight to 43 for validation cohorts, with less than half of the studies including 50 or above cases. All studies included a control group for comparison of methylation patterns. All but one study [19] included a healthy control group, and the cohort sizes had a large span from seven to 1587 individuals. Thirteen of the studies also included a benign control group with numbers ranging from five to 119, with one of the studies only including the benign control group [19]. In one study, the case and control data appear to be reused from another study by the same author [30, 33], and in another study, the healthy control group data appear to be reused from another study by the same author [31, 35] without any of the studies reporting that data were previously published.

The most reported histological subtype of OC was serous, followed by endometrioid and mucinous﻿ (Table 1), corresponding to serous being the most abundant subtype [2]. Four studies did not report which subtypes were studied. Advanced stages (III–IV) were included in all studies with available staging data, and 19 of these studies also included early stages (I–II) (Table 1).

The initial target gene selection was for most studies based on previous research and literature review, with only seven studies identifying target genes from a global methylation screen. Seventeen studies investigated methylation of promoter regions, three studies examined either multiple regions, including promoter regions, or other regions of the gene, e.g., exons, and nine studies did not report which region of the gene was investigated.

### Preanalytical treatment of samples

Preanalytical sample handling is crucial for cfDNA analysis, and multiple factors can affect downstream results, including sample material, blood collection tube, volume, centrifugation regimen, sample storage, DNA extraction method, and pretreatment of DNA [46, 47].

Serum and plasma were used as sample material for cfDNA extraction in 15 and 14 studies, respectively, with plasma being the most abundantly used in the more recent studies (Table 2).

**Table 2:** Preanalytical factors for methylation analysis of the included studies

Author (et al.)	Sample type	Volume (ml)	Selection of samples	Blood collection tubes	Blood sample processing and sample storage	DNA extraction method	Bisulfite conversion/Pretreatment of DNA
de Caceres [18]	Serum	1.5	Retrospective	N/A	N/A	Proteinase K/SDS at 37 °C overnight Phenol/chloroform	Sodium bisulfite conversion
Su [19]	Serum	1	N/A	N/A	3000×*g*, 10 min	QIAamp DNA Blood Mini Kit (QIAGEN)	EZ DNA Methylation Kit (Zymo Research) Modified DNA stored at − 80 °C until analysis
Melnikov [20]	Plasma	0.2	Retrospective	N/A	N/A	DNAzol reagent (Molecular Research Center)	Methylation-sensitive restriction enzyme treatment
BonDurant [21]	Serum	0.5	Retrospective	N/A	N/A	Proteinase K/SDS at 50 °C for 48 h Phenol/chloroform (twice)	Conventional sodium bisulfite method Modified DNA stored at 4 °C until analysis
Campan [22]	Serum	0.1	N/A	N/A	Coagulated for 1–4 h, RT 200×*g*, 10 min, RT Stored at − 80 °C	QIAamp UltraSens Virus Kit (QIAGEN)	EZ DNA Methylation Kit (Zymo Research)
Häfner [23]	Serum	0.7	N/A	N/A	N/A	NucleoSpin Plasma XS Kit (Macherey–Nagel)	MethylAmp DNA Modification Kit (Epigentek) Modified DNA stored at − 20 °C until analysis
Liggett [24]	Plasma	0.2	N/A	EDTA	Processed within 2 h after blood draw 1. 2600×*g*, 10 min, 4 °C 2. 2600×*g*, 10 min, 4 °C Stored at − 80 °C	DNAzol BD Reagent (ThermoFisher Scientific)	Methylation-sensitive restriction enzyme treatment
Dong [25]	Serum	0.5	Prospective	N/A	Coagulated for 2 h at RT 3000×*g*, 10 min Stored at − 80 °C	Phenol/chloroform	CpGenome DNA Modification Kit (Chemicon)
Wang [26]	Serum	N/A	Retrospective	N/A	N/A	QIAamp DNA Blood Mini Kit (Qiagen)	EpiTect Bisulfite Kit (QIAGEN)
Zhang [27]	Serum	0.2	Retrospective/prospective	Vacutainer separating gel procoagulant tubes	3500×*g* , 5 min, 4 °C Stored at − 80 °C	QIAamp MinElute Virus Spin Kit (QIAGEN)	EpiTect Bisulfite Kit (QIAGEN)
Wu [28]	Plasma	N/A	N/A	N/A	N/A	Proteinase K Phenol/chloroform	Conventional sodium bisulfite method Modified DNA stored at − 80 °C until analysis
Zhou [29]	Serum	0.8	N/A	N/A	N/A Stored at − 70 °C	QIAamp DNA Mini Kit (QIAGEN)	Methylation-sensitive restriction enzyme treatment
Wang [30]	Serum	N/A	N/A	N/A	3500 rpm/min, 5 min, 4 °C Stored at − 80 °C	QIAamp DNA Mini Kit (QIAGEN)	N/A
Giannopoulou [31]	Plasma	2	N/A	EDTA	1500×*g* , 10 min Stored at − 80 °C	QIAamp Circulating Nucleic Acid Kit (QIAGEN)	EZ DNA Methylation-Gold Kit (Zymo Research) Modified DNA stored at − 70 °C until analysis
Swellam [32]	Serum	3	N/A	N/A	4000×*g* , 10 min Stored at − 80 °C	QIAamp DNA Blood Mini Kit (QIAGEN) DNA stored at − 80 °C until bisulfite conversion	EpiTect Fast Bisulfite Kit (QIAGEN)
Wang [33]	Serum	0.2	N/A	N/A	3500×*g* , 5 min, 4 °C Stored at − 80 °C	QIAamp DNA Blood Mini Kit (QIAGEN)	EpiTect Bisulfite Kit (QIAGEN)
Widschwendter [34]	Serum	1	Prospective	VACUETTE® Z Serum Sep Clot Activator tubes	Processed up to 12–28 h after blood draw 3000 rpm, 10 min Stored at − 80 °C	Extracted at GATC Biotech (Konstanz, Germany)	Sodium bisulfite conversion at GATC Biotech (Konstanz, Germany)
Giannopoulou [35]	Plasma	2	N/A	EDTA	1500×*g*, 10 min Stored at − 80 °C	QIAamp Circulating Nucleic Acid Kit (QIAGEN)	EZ DNA Methylation-Gold Kit (Zymo Research) Modified DNA stored at − 70 °C until analysis
Dvorská [36]	Plasma	N/A	N/A	N/A	N/A	QIAamp DSP Virus Kit (QIAGEN)	Epitect Bisulfite Kit (QIAGEN) Modified DNA stored at − 20 °C until analysis
Kumar [37]	Plasma	N/A	N/A	EDTA	3000 rpm, 10 min Stored at − 80 °C	Proteinase K/SDS at 37 °C overnight Phenol/chloroform DNA stored at − 20 °C until bisulfite conversion	EZ DNA Methylation Lightening Kit (Zymo Research)
Liu [11]	Plasma	10	Prospective	Cell-free DNA BCT (Streck)	Processed within 1–5 days after blood draw	QIAamp Circulating Nucleic Acid Kit (QIAGEN) Automated MagMax Kit (ThermoFisher Scientific)	EZ-96 DNA Methylation Kit (Zymo Research)
Miller [38]	Plasma	1–3.5	N/A	EDTA	N/A Stored at − 80 °C	NeoGeneStar Cell Free DNA Purification Kit (NeoGeneStar)	EZ DNA Methylation Lightening Kit (Zymo Research)
Singh [39]	Serum	1	N/A	BD Vacutainer® separating gel procoagulation tubes	Processed immediately after blood draw 3500×*g*, 10 min, 4 °C Stored at − 80 °C	Proteinase K/SDS MagMax Cell-Free DNA Magnetic beads extraction (ThermoFisher Scientific) DNA stored at − 20 °C until bisulfite conversion	Premium Bisulfite Kit (Diagenode) Modified DNA stored at − 20 °C until analysis
Faaborg [40]	Plasma	1.6–4.1	Retrospective/ prospective	EDTA	Processed within 4 h after blood draw 1. 2000×*g*, 10 min 2. 10,000×*g*, 10 min (after thawing of plasma) Stored at − 80 °C	QIASymphony DSP Circulating DNA (QIAGEN)	EZ DNA Methylation Lightning Kit (Zymo Research)
Miller [41]	Plasma	1.5–4	Retrospective	N/A	N/A	NeoGeneStar Cell Free DNA Purification Kit (NeoGeneStar)	EZ DNA Methylation Lightning Kit (Zymo Research) Modified DNA stored at − 20 °C until analysis
Singh [42]	Serum	1	N/A	BD Vacutainer separating gel procoagulation tubes	1. 3500×*g*, 1 min, 4 °C 2. 3500×*g*, 1 min, 4 °C Stored at − 80 °C	MagMax Cell-Free DNA Isolation Kit (ThermoFisher Scientific)	Premium Bisulfite Kit (Diagenode) Modified DNA stored at − 20 °C until analysis
Tserpeli [43]	Plasma	2	N/A	EDTA	1500×*g*, 10 min Stored at − 80 °C	QIAamp Circulating Nucleic Acid Kit (QIAGEN)	EZ DNA Methylation Gold Kit (Zymo Research) Modified DNA stored at − 70 °C until analysis
Marinelli [44]	Plasma	3–6	Retrospective	EDTA LBgard® Blood Tubes (Biomatrica)	N/A	QIAamp DNA Blood Kit (QIAGEN)	EZ-96 DNA Methylation Kit (Zymo Research)
Tomeva [45]	Plasma	4	Prospective	EDTA	Processed within 1 h after blood draw 1. 2000×*g*, 10 min, 4–8 °C 2. 16,000 × g, 10 min, 4–8 °C Stored at − 80 °C until shipment, − 20 °C after arrival	MagMAX Cell-Free DNA Isolation Kit (ThermoFisher Scientific) KingFisher Duo Prime Magnetic Particle Processor (ThermoFisher Scientific) DNA stored at − 20 °C until further treatmet	Separation of methylated/unmethylated using MethylMiner™ Methylated DNA Enrichment Kit (Invitrogen, ThermoFisher)

*BCT* blood collection tube, *EDTA* ethylenediamine tetraacetic acid, *H* hours, *Min* minutes, *N/A* not available, *Rpm* revolutions per minute, *RT* room temperature

Processing of plasma and serum is an important step for the yield of cfDNA and for avoiding contamination with high molecular DNA from lysis of cells in the blood sample [46, 47]. To minimize cell lysis, blood samples should be processed within a few hours after the blood draw [46, 47], or blood samples should be drawn in cfDNA-specialized blood collection tubes (BCT) that can stabilize cells and cfDNA for up to two weeks [46–48]. Only six studies reported the time from blood draw to processing, and of these, all, but one study, processed the samples within the recommended time, which is four hours for EDTA BCTs and 7–14 days for cell-free DNA BCTs from Streck [11, 24, 39, 40]. One study processed samples up to 12–28 h after the blood draw and reported that it led to contamination of high molecular DNA, which was assumed to affect the downstream analysis [34].

Double centrifugation is recommended when working with cfDNA as it can reduce contamination with high molecular DNA [46, 47]. Only four studies used a double centrifugation protocol, with two of the studies including a high-force second centrifugation [40, 45]. Thirteen studies used a single centrifugation regimen with forces ranging from 200×*g* to 4000×*g*, and 12 studies did not report how samples were processed. The input sample volume varied substantially from 0.1 to 10 ml, with nine studies using below 1 ml and 15 studies using 1 ml or above. Five studies did not report the volume of sample material used. The storage conditions of plasma and serum samples were reported in 18 studies, with 16 studies storing samples at − 80 °C, one storing at − 70 °C, and one storing at − 80 °C until shipment of samples and thereafter storage at − 20 °C until analysis.

For an optimal yield of cfDNA, specialized kits or methods are recommended for DNA extraction [46, 47]. The DNA extraction was for 11 studies performed using specialized cfDNA extraction kits, with most studies using Qiagen’s Circulating Nucleic Acid Kit. Three studies used a virus nucleic acid extraction kit, nine studies used a genomic DNA blood kit, five used conventional phenol/chloroform DNA extraction, and one study had a company do the DNA extraction with no available information of the extraction method [34].

For methylation analysis, pretreatment of the DNA is necessary for discrimination between methylated and unmethylated DNA.

Bisulfite conversion is a harsh chemical treatment in which unmethylated cytosines are converted to uracil, and methylated cytosines are conserved, allowing discrimination of methylated and unmethylated DNA [49, 50]. Bisulfite conversion requires a purification and extraction step, which, together with degradation during the treatment, can result in a substantial loss of DNA [49, 50]. Twenty studies used a commercial bisulfite conversion kit, with the most frequently used being kits from Zymo Research and Qiagen (Table 2). Three studies used a conventional bisulfite conversion method, and one study had a company for the bisulfite conversion without any available information on the method used [34].

Three studies used methylation-sensitive restriction enzyme (MSRE) treatment, which is another approach used for discriminating methylated DNA from unmethylated DNA [20, 24, 29]. In MSRE treatment, methylated CpGs are kept intact, whereas unmethylated CpGs are cleaved, leaving only methylated DNA to be analyzed [51].

One study used a method in which they separated methylated DNA from unmethylated with a capture-based kit that uses a methyl-CpG binding protein coupled to Streptavidin magnetic beads [45, 52]. The treatment is less harsh than bisulfite conversions; however, the method only recovers double-stranded DNA, requires a minimum of 5 ng DNA, and yields a total of CpG-methylated DNA of 3–20% of the input mass of DNA [52–54], making the method less favorable when doing DNA methylation analysis of low DNA input samples such as cfDNA from plasma and serum.

One study did not report which type of pretreatment of the DNA was used [33].

### Methylation analysis methods

In Tables 3 and 4, the methylation analysis methods are summarized. The most utilized methods were PCR-based, with 12 studies using methylation-specific PCR (MSP), of which three studies used a nested PCR method. Real-time quantitative MSP (qMSP) was used in seven studies, MSRE PCR was used in three studies, and one study used real-time qPCR. One study used digital MSP, and one used methylation-specific droplet digital PCR. One study used a Target Enrichment Long-probe Quantitative Amplified Signal assay (TELQAS), which is a modification of a quantitative allele-specific real-time target and signal amplification method [44].

**Table 3:** Gene-specific methylation biomarkers investigated in plasma or serum from OC and controls, including methods, methylation frequency, and performance for early diagnosis of OC

Gene	Author (et al.)	Method	Case/control	Meth OC	Meth control	Sensitivity % (95% CI)	Specificity % (95% CI)
*APC*	de Caceres [18]	MSP	50/10^b^/20^h^ (T)	N/A	N/A	N/A	N/A
	Zhang [27]	MSP, Nested multiplex	87/53^b^/62^h^ (T) 39/29^b^ (V)	N/A	N/A	N/A	N/A
	Tomeva [45]	Real-Time qPCR	19/8^h^ (T)	N/A	N/A	N/A	N/A
*BRCA1*	de Caceres [18]	MSP	50/10^b^/20^h^ (T)	N/A	N/A	N/A	N/A
	Melnikov [20]	MSRE PCR/microarray	33/33^h^ (T)	31/33*	17/33*	93.9* (79.8–99.3)	48.5* (30.8–66.5)
	Liggett [24]	MSRE PCR/microarray	30/30^b^/30^h^ (T)	N/A	N/A	N/A	N/A
	Wang [26]	Real-time qMSP	60/30^b^/30^h^ (T)	N/A	N/A	N/A	N/A
	Kumar [37]	MSP	53/12^b^/15^h^ (T)	33/53	6/12^b^ 0/15^h^	62.3* (47.9–75.2)	77.8* (57.7–91.4)
	Tserpeli [43]	Real-time qMSP	84/49/27^h^ (T)	10/82 10/48	0/27	12.2 (6.01–21.3) 20.8 (10.5–35.0)	100 (87.2–100)
*CDH1*	Zhang [27]	MSP, Nested multiplex	87/53^b^/62^h^ (T) 39/29^b^ (V)	N/A	N/A	N/A	N/A
	Dvorská [36]	Pyrosequencing	33/5^b^/9^h^ (T)	N/A	N/A	N/A	N/A
*DAPK*	Häfner [23]	MSP/Sanger sequencing	32/30^b^/20^h^ (T)	13/23 ^†^	5/21^b^ 4/8^h †^	56.5* (34.5–76.8)	69.0* (49.2–84.7)
	Swellam [32]	MSP	90/50^b^/30^h^ (T)	87/90	20/50^b^/0/30^h^	96.7 (90.6–99.3)	75.0 (64.1–84.0)
*HIC1*	Melnikov [20]	MSRE PCR/microarray	33/33^h^ (T)	26/33*	17/33*	78.8* (61.1–91.0)	48.5* (30.8–66.5)
	Singh [39]	Real-time qMSP	45/25^h^ (T)	32/45	0/25	71.1 (55.7–83.6)	100 (86.3–100)
	Singh [42]	Real-time qMSP, Multiplex	45/25^h ■^ (T)	32/45	0/25	71.1 (55.7–83.6)	100 (86.3–100)
*HOXA9*	Singh [39]	Real-time qMSP	45/25^h^ (T)	28/45	0/25	62.2 (46.5–76.2)	100 (86.3–100)
	Faaborg [40]	Methylation-specific Droplet digital PCR	79/64^h^ (T)	47/79	3/64*	59.5 (47.9–70.4)	95.3 (86.9–99.0)
	Singh [42]	Real-time qMSP, Multiplex	45/25^h ■^ (T)	28/45	0/25	62.2 (46.5–76.2)	100 (86.3–100)
*OPCML*	Zhang [27]	MSP, Nested multiplex	87/53^b^/62^h^ (T) 39/29^b^ (V)	N/A	N/A	N/A	N/A
	Zhou [29]	MSRE PCR	45/20^h^ (T)	36/45	0/20	80.0 (65.4–90.4)	100 (83.2–100)
	Wang [30]	MSP, Nested multiplex	71/43^b^/80^h^ (T)	N/A	N/A	N/A	N/A
	Swellam [32]	MSP	90/50^b^/30^h^ (T)	88/90	24/50^b^ 0/30^h^	97.8 (92.2–99.7)	70.0 (58.7–79.7)
	Wang [33]	MSP, Nested	71/43^b^/80^h ‡^ (T)	64/71	10/123*	90.1 (80.7–95.9)	91.9 (85.6–96.0)
*PAX1*	Su [19]	MSP	26/20^b^ (T)	5/26	0/20^b^	19.2* (6.55–39.4)	100* (83.2–100)
	Dvorská [36]	Pyrosequencing	33/5^b^/9^h^ (T)	N/A	N/A	N/A	N/A
*PGR*	Melnikov [20]	MSRE PCR/microarray	33/33^h^ (T)	27/33*	15/33*	81.8* (64.5–93.0)	54.6* (36.4–71.9)
	Liggett [24]	MSRE PCR/microarray	30/30^b^/30^h^ (T)	N/A	N/A	N/A	N/A
*RASSF1A*	de Caceres [18]	MSP	50/10^b^/20^h^ (T)	N/A	N/A	N/A	N/A
	BonDurant [21]	Real-time qMSP	21/7^h^ (T)	18/21	N/A	85.7* (63.7–97.0)	N/A
	Liggett [24]	MSRE PCR/microarray	30/30^b^/30^h^ (T)	N/A	N/A	N/A	N/A
	Zhang [27]	MSP, Nested multiplex	87/53^b^/62^h^ (T) 39/29^b^ (V)	N/A	N/A	N/A	N/A
	Giannopoulou [31]	Real-time qMSP/MS-HRMA	59/51^h^ (T)	15/59	0/51	25.4* (15.0–38.4)	100* (93.0–100)
	Kumar [37]	MSP	53/12^b^/15^h^ (T)	20/53	2/12^b^/0/15^h^	37.7* (24.8–52.1)	92.6* (75.7–99.1)
	Tomeva [45]	Real-Time qPCR	19/8^h^ (T)	N/A	N/A	N/A	N/A
*RUNX3*	Zhang [27]	MSP, Nested multiplex	87/53^b^/62^h^ (T) 39/29^b^ (V)	N/A	N/A	N/A	N/A
	Wang [30]	MSP, Nested multiplex	71/43^b^/80^h^ (T)	N/A	N/A	N/A	N/A
*SFRP5*	Su [19]	MSP	26/20^b^ (T)	4/26	2/20^b^	15.4* (4.36–34.9)	90.0* (68.3–98.8)
	Zhang [27]	MSP, Nested multiplex	87/53^b^/62^h^ (T) 39/29^b^ (V)	N/A	N/A	N/A	N/A
*SOX1*	Su [19]	MSP	26/20^b^ (T)	15/26	3/20^b^	57.7* (36.9–76.7)	85.0 (62.1–96.8)
	Singh [42]	Real-time qMSP/, Multiplex	45/25^h ■^ (T)	24/45	1/25	53.3 (37.9–68.3)	96.0 (79.7–99.9)
*TFPI2*	Zhang [27]	MSP, Nested multiplex	87/53^b^/62^h^ (T) 39/29^b^ (V)	N/A	N/A	N/A	N/A
	Wang [30]	MSP, Nested multiplex	71/43^b^/80^h^ (T)	N/A	N/A	N/A	N/A
*ZNF154*	Miller [38]	DREAMing	26/41^h^ (T) 8/12^h^ (V)	N/A	N/A	65.0 Epiclass/54.0 Mean Meth 91.7 Epiclass/ 83.3 Mean Meth	83.0 Epiclass/63.0 Mean Meth 100 Epiclass/66.7 Mean Meth
	Miller [41]	DREAMing	38/20^h^ (T)	33/38*	4/20*	86.8 (71.9–95.6)	80.0 (56.3–94.3)

Only gene-specific methylation biomarkers reported in more than one study are included. 95% exact confidence intervals (CI) have been calculated when data were available

*b* benign, *bl* borderline tumor, *h* healthy, *Meth* methylated, *MSP* methylation-specific-PCR, *MSRE* methylation-sensitive restriction enzyme, *MS-HRMA* methylation-sensitive high resolution melting analysis, *N/A* not available, *PCR* polymerase chain reaction, *qMSP* quantitative methylation-specific PCR, *qPCR* quantitative PCR, *TELQAS* Target Enrichment Long-probe Quantitative Amplified Signal, *T* training/test cohort, *V* validation cohort

^*^Extrapolated calculations based on available data

^†^The number of included methylation frequencies varies to the cohort size as only samples with *beta-actin* amplification were analyzed further

^‡^Appear to be use of cohort and/or data from Wang et al. [30]

^■^Reuse of data from Singh et al*.* [39]

**Table 4:** Methylation panels investigated in plasma or serum from OC and controls for early diagnosis of OC, including information on methods, methylation frequency, and performance

Methylation panel	Author (et al.)	Method	Case/control	Meth OC	Meth control	Sensitivity % (95% CI)	Specificity % (95% CI)
*APC,APKinase, BRCA1, p14ARF, p16INK4a, RASSF1A*	de Caceres [18]	MSP	50/10^b^/20^h^ (T)	41/50	0/10^b^ 0/20^h^	82.0 (68.6–91.4)	100* (88.4–100)
*SOX, PAX1, LMX1A, SFRP1, SFRP2, SFRP5*	Su [19]	MSP	26/20^b^ (T)	19/26*	9/20*	73.1 (52.2–88.4)	55.0 (31.5–76.9)
*SOX, PAX1, SFRP1*	Su [19]	MSP	26/20^b^ (T)	19/26*	5/20*	73.1 (52.2–88.4)	75.0 (50.9–91.3)
*BRCA1, HIC1, PAX5, PGR, THBS1*	Melnikov [20]	MSRE PCR/microarray	33/33^h^ (T)	28/33*	13/33*	85.1 (68.1–94.9)	61.1 (42.1–77.1)
*CALCA, EP300, RASSF1A*	Liggett [24]	MSRE PCR/microarray	30/30^h^ (T)	27/30*	4/30*	90.0 (73.5–97.9)	86.7 (69.3–96.2)
*PGR, RASSF1A*	Liggett [24]	MSRE PCR/microarray	30/30^b^ (T)	22/30*	6/30*	73.3 (54.1–87.7)	80.0 (61.4–92.3)
*APC, CDH1, OPCML, RASSF1A, RUNX3, SFRP5, TFPI2*	Zhang [27]	MSP, Nested multiplex	87/53^b^ (T) 39/29^b^ (V)	78/87 36/39	5/53 5/29	89.7 (81.3–95.2) 92.3 (79.1–98.4)	90.6 (79.3–96.9) 82.8 (64.2–94.2)
*OPCML, RUNX3, TFPI2*	Wang [30]	MSP, Nested multiplex	71/43^b^/80^h^ (T)	64/71	11/123*	90.1 (80.7–95.9)	91.1 (84.6–95.5)
*COL23A1, C2CD4D, WNT6*	Widschwendter [34]	Targeted NGS	29/119^b^/21^h^ (T) 48/154^b^/41^h^ (V)	12/29 28/48	13/140 16/195	41.4 (23.5–61.1) 58.3 (43.2–72.4)	90.7 (84.6–95.0) 91.8 (87.0–95.2)
*CDH1, PAX1, PTEN, RASSF1*	Dvorská [36]	Pyrosequencing	33//9^h^ (T)	30/33*	4/9*	91.0 (75.7–98.1)	56.0 (21.2–86.3)
Panel of 103,456 distinct regions and 1,116,720 CpGs	Liu [11]	Targeted NGS	27/1521^h^ (T) 12/610^h^ (V)	26/27 12/12	3/1521* 4/610*	96.0 (81.0–99.9) 100 (73.5–100)	99.8 (99.4–100) 99.3 (98.3–99.8)
*HIC1, HOXA9*	Singh [39]	Real-time qMSP	45/25^h^ (T)	40/45	0/25	88.9 (76.0–96.3)	100 (86.3–100)
*HIC1, HOXA9*	Singh [42]	Real-time qMSP, Multiplex	45/25^h ■^ (T)	40/45	0/25	88.9 (76.0–96.3)	100 (86.3–100)
*HOXA9, SOX1*	Singh [42]	Real-time qMSP, Multiplex	45/25^h ■^ (T)	30/45	1/25	66.7 (51.1–80.0)	96.0 (79.7–99.9)
*HIC1, SOX1*	Singh [42]	Real-time qMSP, Multiplex	45/25^h ■^ (T)	36/45	1/25	80.0 (65.4–90.4)	96.0 (79.7–99.9)
*AGRN, BCAT1, CAPN2, CDO1, CELF2, FAIM2, GPRIN1, GYPC, RIPPLY3, SRC, SIM2*	Marinelli [44]	TELQAS	91/91^h^ (T)	72/91*	4/91*	79.0 (69.3–86.9)	96.0 (89.1–98.8)

95% exact confidence intervals (CI) have been calculated for all methylation panels

*b* benign, *CI* confidence intervals, *h* healthy, *Meth* methylated, *MSP* methylation-specific PCR, *MSRE* methylation-sensitive restriction enzyme, *N/A* not available, *PCR* polymerase chain reaction, *qMSP* quantitative methylation-specific PCR, *TELQAS* Target Enrichment Long-probe, Quantitative Amplified Signal, *T* training/test cohort, *V* validation cohort

^*^Extrapolated calculations based on available data

^■^Reuse of data from Singh et al*.* [39]

Multiple methylated regions were investigated by several studies using sequencing approaches. Two studies used targeted NGS, one study used pyrosequencing, one used MSRE microarray, and one study used Sanger sequencing. Methylation-sensitive high-resolution melt analysis (MS-HRMA) was used by one study, and two studies used a method called DREAMing, which employs some of the principles of MS-HRMA but is directly quantitative and highly sensitive [55].

### Gene-specific methylation biomarkers

In total, methylation of 60 genes was investigated by the 29 studies included. Genes that were investigated in more than one study are summarized in Table 3, and genes that were investigated in only one study are summarized in Additional file 3. The most investigated genes were *RASSF1A*, *BRCA1*, *OPCML*, *APC*, *HIC1*, and *HOXA9*, all being tumor-suppressor genes [56].

Seven studies investigated *RASSF1A*, with three of the studies reporting or having data available for the calculation of performance measurements for the gene as a single biomarker. The sensitivity of *RASSF1A* as a single biomarker was found to be 25.4% (95% exact confidence intervals (CI) 15.0–38.4), 37.7% (95% CI 24.8–52.1), and 85.71% (95% CI 63.7–97.0) [21, 31, 37], with corresponding specificities of 100% (95% CI 93.0–100), 92.6% (95% CI 75.7–99.1), and not reported for the study with the highest sensitivity (Table 3). *RASSF1A* as a single methylation biomarker of OC is inconclusive as very different performances are reported.

*BRCA1* was investigated in six studies, but only three studies had available data for evaluating the gene as a single biomarker for OC. The sensitivity was reported to be 12.2% (95% CI 6.01–21.3)/20.8% (95% CI 10.5–35.0), 62.3% (95% CI 47.9–75.2), and 93.9% (95% CI 79.8–99.3), with corresponding specificities of 100% (95% CI 87.2–100), 77.8% (95% CI 57.7–91.4), and 48.5% (95% CI 30.8–66.5), respectively [20, 37, 43]. For *BRCA1,* the performance varied substantially, and *BRCA1* either had a too low sensitivity or a too low specificity to be a single biomarker for OC.

Five studies investigated the methylation of *OPCML*, with three of the studies reporting performance measurements. *OPCML* had a rather high sensitivity of 80.0% (95% CI 65.4–90.4), 90.1% (95% CI 80.7–95.9), and 97.8% (95% CI 92.2–99.7) in the three studies [29, 32, 33]. The corresponding specificities were also high, with 100% (95% CI 83.2–100), 70.0% (95% CI 58.7–79.7), and 91.9% (95% CI 85.6–96.0), respectively. *OPCML* as a biomarker of OC looks promising; however, none of the studies which reported performance have validated their findings.

*HIC1* was investigated in three studies, with one of the studies reusing data yielding identical performance measurements in two of the three studies. The sensitivity of *HIC1* was 71.1% (95% CI 55.7–83.6), 71.1% (95% CI 55.7–83.6), and 78.8% (95% CI 61.1–91.0), with corresponding specificities of 100% (95% CI 86.3–100), 100% (95% CI 86.3–100), and 48.5% (95% CI 30.8–66.5) [20, 39, 42].

*HOXA9* was also investigated by three studies, with one of the studies reusing data as for *HIC1*. The sensitivity of *HOXA9* was 62.2% (95% CI 46.5–76.2), 62.2% (95% CI 46.5–76.2), and 59.5% (95% CI 47.9–70.4) [39, 40, 42], with a specificity of 100% (95% CI 86.3–100), 100% (95% CI 86.3–100), and 95.3% (95% CI 86.9–99.0), respectively. Although the sensitivity for *HIC1* and *HOXA9* is more consistent between the studies and higher than for some of the other genes, the sensitivities are too low for a diagnostic biomarker of OC. In addition, the specificity of *HIC1* differs substantially between the studies. *APC* was investigated in three studies, with none of the studies reporting performance measurements of the gene.

*ZNF154* was investigated as a single biomarker of OC in two studies performed by the same author [38, 41]. *ZNF154* had relatively high sensitivity and specificity in both studies (Table 3), and one of the studies developed a classifier that performed better than using mean methylation values [38].

### Methylation panels

Twelve studies investigated methylation panels consisting of two or more genes or distinct regions and CpGs (Table 4). In total, 15 gene-specific methylation panels and one panel of 103,456 distinct regions and 1,116,720 CpGs were reported, all with available performance measurements. Overall, combining two or more genes in a gene-specific methylation panel increased sensitivity and specificity for the detection of OC compared to using only a single gene (Tables 3, 4). The sensitivity ranged from 41.4 to 100%, with 12 of the 16 methylation panels having a sensitivity above 75%. The specificity varied from 55.0 to 100%, with seven methylation panels having a sensitivity above 95%.

The best-performing methylation panel was a panel of 103,456 distinct regions and 1,116,720 CpGs reported by Liu et al*.* [11]. The sensitivity was 96.0% (95% CI 81.0–99.9) and 100% (95% CI 73.5–100) in the training and validation cohort, respectively, and the corresponding specificities were reported to be 99.8% (95% CI 99.4–100) and 99.3% (95% CI 98.3–99.8). The study only included a small number of OC cases, with 27 cases in the training cohort and 12 cases in the validation cohort. This is important to note as fewer cases make results less robust. The methylation panel of *APC*, *CDH1*, *OPCML*, *RASSF1A*, *RUNX3*, *SFRP5*, and *TFPI2* reported by Zhang et al*.* [28] also performed well in the validation cohort with a sensitivity of 92.3% (95% CI 79.1–98.4) and a specificity of 82.8% (95% CI 64.2–94.2). However, the validation cohort only included six OC cases making performance results ambiguous. Wang et al*.* [30] reported a methylation panel consisting of three of the genes reported by Zhang et al*.* [28]. The panel consisted of *OPCML*, *RUNX3*, and *TFPI2* and had a sensitivity of 90.1% (95% CI 80.7–95.9) and a specificity of 91.1% (95% CI 84.6–95.5). The study included a cohort size that was larger, with 71 OC cases, and even though the combination of these three genes seems promising as a methylation panel to diagnose OC, the results have not been validated, and the specificity is borderline acceptable in the context of a diagnostic or screening tool yielding 9% false positives cases. The methylation panel of *CALCA*, *EP300*, and *RASSF1A* was reported by Liggett et al*.* [24] with a sensitivity of 90.0% (95% CI 73.5–97.9) and a specificity of 86.7% (95% CI 69.3–96.2). The study only included 30 OC cases and had a specificity below 90%, leaving this panel less likely to be useful as a diagnostic or screening tool for OC.

## Discussion

In this systematic review, 29 original research articles investigating DNA methylation biomarkers in ctDNA from serum or plasma for diagnosis of OC were identified. We have summarized the differences in both gene-specific methylation biomarkers and methylation panels between OC patients, patients with benign conditions of the ovaries, and healthy females from the included studies.

The most investigated gene-specific methylation biomarkers were *RASSF1A*, *BRCA1*, and *OPCML*, all being tumor-suppressor genes previously reported to be involved in tumorigenesis and methylated in multiple solid cancers [57–60]. It was consistently reported that *RASSF1A*, *BRCA1*, and *OPCML* had higher methylation frequencies in OC compared to patients with benign disease of the ovaries and/or healthy females (Table 3).

*OPCML* was the single gene-specific methylation biomarker with the best performance measures (Table 3). The highest corresponding sensitivity of 97.8% (95% CI 92.2–99.7) and specificity of 91.9% (95% CI 85.6–96.0) were reported by Wang et al. [33] for methylated *OPCML* to discriminate between OC and benign disease of the ovaries as well as healthy females in serum using a nested MSP approach.

Several of the reported gene-specific methylation biomarkers were only included in one or few studies, and performance measures were lacking for many of them, which renders our ability to conduct an overall comparison of gene-specific methylation biomarkers.

However, from the gene-specific methylation biomarkers that did report performance measures, it was evident that they did not perform as well as the methylation panels (Tables 3, 4).

The methylation panel that performed best was a panel of 103,456 distinct regions and 1,116,720 CpGs reported by Liu et al*.* using a targeted NGS approach [11]. The panel had a sensitivity of 96.0% (95% CI 81.0–99.9) and a specificity of 99.8% (95% CI 99.4–100) to discriminate OC from healthy controls in the training cohort, and a sensitivity of 100% (95% CI 73.5–100) and a specificity of 99.3% (95% CI 98.3–99.8) in the validation cohort. The wide range of methylation sites investigated could be a contributing factor to the panel being the best to discriminate OC from healthy controls. A methylation panel of three biomarkers, *OPCML, RUNX3,* and *TFPI2,* investigated by Wang et al*.* [30] also performed well with a sensitivity of 90.1% (95% CI 80.7–95.9) and a specificity of 91.1% (95% CI 84.6–95.5) to discriminate OC from patients with benign disease of the ovaries and healthy females in serum using a nested multiplex MSP approach. Similar to what has previously been reported for *OPCML*, *RUNX3* and *TFPI2* have also been reported to be methylated, thereby promoting tumorigenesis, in other solid cancers, mainly of the digestive tract [61–63].

It was not evident how the wide range of study designs, pretreatment factors, as well as methods affected the performance of the methylation biomarkers. The best-performing methylation panel from Liu et al*.* [11] was designed for ctDNA methylation analysis, and preanalytical parameters had been taken into consideration, which could be a contributing factor to the better performance of this panel. The study used 10-ml plasma collected in cell-free DNA BCTs from Streck and extracted cfDNA with a kit specialized for cfDNA extraction. On the contrary, the methylation panel of *OPCML, RUNX3,* and *TFPI2* from Wang et al*.* [30] and the best-performing single gene-specific methylation biomarker *OPCML* investigated by Wang et al*.* [33] used serum, with Wang et al*.* [33] using only 0.2 ml and Wang et al*.* [30] not specifying volume used, and cfDNA was extracted with a kit developed for whole blood DNA extraction.

The amount of cfDNA extracted from plasma or serum depends on the DNA extraction method used [64–66]. Kits for DNA extraction from whole blood are developed for DNA with a high molecular weight, and their use in cfDNA extraction can lead to suboptimal yield [64–66]. Only 11 of the 29 studies used kits developed for cfDNA extraction. It cannot be excluded that this could have led to low cfDNA yield, which would be an obstacle for detection of methylation biomarkers in plasma and serum and could have contributed to the lack of sensitivity of some of the biomarkers studied.

Moreover, it has been shown that although the concentration of cfDNA in serum tends to be higher than in plasma, serum has a lower detection rate of tumor-derived cfDNA [67, 68]. This is a result of tumor-derived cfDNA being diluted in higher levels of non-tumor cfDNA and can be contaminated with high molecular weight DNA providing limitations on detection of low-frequency ctDNA alleles [67, 68].

The amount of converted cfDNA being available for methylation analysis can be considerably affected by the choice of bisulfite conversion kits [69]. However, we could not establish an obvious effect of kits and methods used for bisulfite conversion, despite a previous study reporting recovery rates ranging from 22% for the poorest and 66% for the best-performing bisulfite conversion kits [69].

Although many of the included studies use similar methylation analysis methods, e.g., MSP, the comparison of results can be influenced by the heterogeneousness of assay design and efficiency as MSP targets specific CpGs. For instance, the sensitivity of *RASSF1A* as a gene-specific methylation biomarker differed considerably in a study by BonDurant et al*.* [21] compared to a study by Giannopoulou et al*.* [31] (Table 3), although both studies used real-time qMSP. The studies used assays that were designed for different CpGs, and this could explain the considerable variation in sensitivity observed between the two studies. The difference in sensitivity could also be a consequence of a large variation in case/controls numbers included in the two studies, with BonDurant et al*.* [21] only including 21 OC and seven controls compared to 59 OC and 51 controls included in Giannopoulou et al*.* [31].

In the studies included in this review, case numbers were relatively low, with no study including more than 91 OC cases. The cohort sizes varied substantially between the studies, which complicate the comparison and evaluation of the methylation biomarkers. The case cohorts predominately consisted of patients with advanced stage, although 19 studies also included stage I and II patients. It could be expected that the predominance of advanced stage will affect the discrimination abilities of the methylation analysis in a direction of better discrimination for advanced stage patients. The patients with advanced stages often present with symptoms making this patient group easier to diagnose with the standard diagnostic tools available compared to patients with early-stage OC as these most often are asymptomatic. A study by Jensen et al*.* [70] observed that a panel of methylation biomarkers had lower sensitivity in asymptomatic colorectal cancer patients compared to symptomatic patients. The sensitivity was markedly reduced for early-stage asymptomatic patients, which was suggested to be a consequence of some asymptomatic cancers shedding less ctDNA than symptomatic patients, highlighting the importance of evaluating the methylation biomarkers in asymptomatic early-stage patients.

Discriminating patients with benign disease of the ovaries from OC patients is clinically important as patients with benign disease often will be suspected to have OC. Therefore, the inclusion of a benign control group to obtain methylation biomarkers that can discriminate between OC and benign disease can have value for the clinical utility of the biomarkers. Only 13 studies included a benign control group, but it was not evident that the inclusion of this control group affected the performance of the methylation biomarkers and panels.

The multiplicity of sample pretreatment and methylation analysis methods used in the included studies highlights the lack of standard agreements for methylation analysis of cfDNA. Only a few studies took factors that affect cfDNA analysis into consideration when interpreting and reporting results. For instance, Widschwendter et al*.* [34] reported that their results could have been affected by a contamination of high molecular DNA due to prolonged time from blood draw to sample processing. A rationale of why so few studies considers the factors affecting methylation analysis of cfDNA is the 18-year age gap between the first and the latest study included in this review. The methodology has developed substantially during this period with, e.g., qPCR, digital PCR, and NGS, as well as our understanding of cfDNA and the factors influencing it. Therefore, it cannot be precluded that the methylation biomarkers investigated in the earliest studies might perform differently with the current methylation analysis methods and with sample pretreatment designed for cfDNA analysis.

Only 20 studies reported performance measures or the numbers of methylated case/controls necessary to calculate the sensitivity and specificity of gene-specific methylation biomarkers and panels, and therefore, not all methylation biomarkers reported as potential biomarkers for OC were evaluated in this review. It cannot be excluded that some of these biomarkers can be promising and can have potential for further investigation. In addition, of the studies that reported performance measures, only a few validated the performance in a validation cohort. Without an external cohort for validation, the conclusiveness of the performance results will be affected, calling for more investigation of the biomarkers to subtract more conclusive remarks hereof.

In this review process, some potential limitations must be considered. The studies were identified from searches in two widely used databases as well as cross-referencing of both bibliography from reviews and the included articles, but the risk of having missed relevant studies for inclusions is present. By excluding non-English articles, there is also a risk of having excluded relevant studies published in non-English.

The main limitation of this review is the limited possibility of conducting a standardized summary of the results due to the diversity and lack of performance measures of the included studies. This also inhibited our possibility of conducting a meta-analysis, which then precluded us from making robust conclusions. However, this systematic review provides an overview of all the methylation biomarkers investigated for early diagnosis and detection of OC in plasma and serum and demonstrates the potential utility of methylation analysis of cfDNA in early diagnosis of OC.

## Conclusion

In summary, this review displays that panels of methylation biomarkers performed better than single gene-specific methylation biomarkers. One methylation panel had a better performance in discriminating OC from healthy and could be a promising potential methylation panel used as a diagnostic tool, although the inclusion of only few OC cases limits the conclusiveness of this study. The methylation panel, as well as other promising methylation biomarkers highlighted in this review, need validation in large, prospective cohorts with early-stage asymptomatic OC patients to assess the true diagnostic value in a clinical setting.

The field of methylation analysis of ctDNA requires standardization of preanalytical factors, including detailed reporting of sample pretreatment and methods used, and sensitive methods as the heterogeneity of the included studies complicates the evaluation of the diagnostic potential of promising biomarkers.

## Supplementary Information

**Additional file 1: Table S1**: Search string with search terms in PubMed and Embase.

**Additional file 2: Table S2**: Quality assessment of included studies using the Newcastle-Ottawa Scale.

**Additional file 3: Table S3**: Gene-specific methylation biomarkers investigated in plasma or serum from OC and controls reported in only one study.

## Data Availability

The data supporting the analysis and conclusions of this article are included within the article and its Additional information files. The review was not registered.

## Abbreviations

BC-OC: Ovarian cancers subsequent to breast cancer
CA-125: Cancer Antigen 125
ctDNA: Circulating tumor DNA
cfDNA: Cell-free DNA
CpG: Cytosine–phosphate–guanine
EDRN: The Early Detection Research Network
EDTA: Ethylenediamine tetraacetic acid
LMP: Low malignant potential
MS-HRMA: Methylation-sensitive high-resolution melt analysis
MSP: Methylation-specific-PCR
MSRE: Methylation-sensitive restriction enzyme
N/A: Not available
OC: Epithelial ovarian cancer
PRISMA: Preferred Reported Items for Systematic Reviews and Meta-Analysis
qMSP: Real-time quantitative MSP
RT: Room temperature
TELQAS: Target Enrichment Long-probe Quantitative Amplified Signal assay
