# Supplementary material for: Early diagnosis of ovarian cancer based on methylation profiles in peripheral blood cell-free DNA: a systematic review
Source: Clin Epigenetics. 2023 Feb 14;15:24. doi: 10.1186/s13148-023-01440-w (PMC9926627; doi:10.1186/s13148-023-01440-w)
Supplement: Supplementary file 1 — Additional file 1: Table S1: Search string with search terms in PubMed and Embase. [file 13148_2023_1440_MOESM1_ESM.docx]

# **Additional file 1**

**Table Additional file 1:** Search string with search terms in PubMed and Embase. The search string was established by combining each search term within each search group with ‘’OR’’ and then combining the three search groups with ‘’AND’’. No filters were applied for the PubMed search. Embase was set to exclude conference abstract, editorials, letters, notes, and short surveys.

| **Database** | **Search Groups** | | |
| --- | --- | --- | --- |
|  | **1. Early diagnosis** | **2. Ovarian cancer** | **3. Methylation biomarker/cfDNA/ctDNA** |
| PubMed | Early diagnosis [MeSH] | Ovarian neoplasms [MeSH] | (Methylation[MeSH] |
|  | Early diagnosis[text word] | Ovarian neoplasms[text word] | DNA Methylation[MeSH] |
|  | Cancer screening[text word] | Ovarian cancer[text word] | Methylat*[text word] |
|  | Diagnos*[text word] | High grade serous[text word] | Hypermethyl*[text word] |
|  | Early detect*[text word] | HGSC[text word] | Epigenetic[text word] |
|  |  | Cystadenocarcinoma, Serous [MeSH] | Epigenomics[MeSH] |
|  |  | Serous epithelial[text word] | Circulating Tumor DNA[MeSH] |
|  |  | Serous cystadenoma[text word] | ctDNA*[text word] |
|  |  | (ovary[text word] OR ovari*[text word])  AND  (cancer[text word] OR carcinoma*[text word] OR neoplasm*[text word] OR tumor*[text word] OR tumour*[text word]) | Circulating tumor DNA[text word] |
|  |  | Fallopian Tube Neoplasms[MeSH] | Circulating tumour DNA[text word] |
|  |  | (fallopian tube*[text word] OR tubal[text word] OR oviduct[text word] OR tuba[text word]  AND  (cancer[text word] OR carcinoma*[text word] OR neoplasm*[text word] OR tumor*[text word] OR tumour*[text word]) | Cell-Free Nucleic Acids[MeSH] |
|  |  |  | cfDNA*[text word] |
|  |  |  | Cell free DNA[text word] |
|  |  |  | Circulating cell free tumor DNA[text word] |
|  |  |  | Circulating cell free tumour DNA[text word] |
|  |  |  | ccfDNA[text word] |
|  |  |  | Liquid biopsy[MeSH] |
|  |  |  | Liquid biops*[text word] |
|  |  |  | Cell free nucleic acid*[text word] |
| Embase | ‘Early diagnosis’/exp | (‘ovary tumor’/exp | Methylation/exp |
|  | ‘Early diagnosis’:ti,ab,kw | ‘Ovarian neoplasms’:ti,ab,kw | ‘DNA Methylation’/exp |
|  | ‘Cancer screening‘:ti,ab,kw | ‘Ovary cancer’/exp | Methylat*:ti,ab,kw |
|  | Diagnos*:ti,ab,kw | ‘High grade serous’:ti,ab,kw | Hypermethyl*:ti,ab,kw |
|  | ‘Early detect*’:ti,ab,kw | HGSC:ti,ab,kw | Epigenetic:ti,ab,kw |
|  |  | cystadenocarcinoma/exp | Epigenetics/exp |
|  |  | ‘Serous cystadenoma’:ti,ab,kw | ‘Circulating Tumor DNA’/exp |
|  |  | ‘Serous epithelial’:ti,ab,kw | ctDNA*:ti,ab,kw |
|  |  | (ovary:ti,ab,kw OR ovari*:ti,ab,kw)  AND  (cancer:ti,ab,kw OR carcinoma*:ti,ab,kw OR neoplasm*:ti,ab,kw OR tumor*:ti,ab,kw OR tumour*:ti,ab,kw) | ‘Circulating tumor DNA’:ti,ab,kw |
|  |  | ‘uterine tube tumor’/exp | ‘Circulating tumour DNA’:ti,ab,kw |
|  |  | (fallopian tube*:ti,ab,kw OR tubal:ti,ab,kw OR oviduct:ti,ab,kw OR tuba:ti,ab,kw )  AND  (cancer:ti,ab,kw OR carcinoma*:ti,ab,kw OR neoplasm*:ti,ab,kw OR tumor*:ti,ab,kw OR tumour*:ti,ab,kw) | ‘Circulating free DNA’/exp |
|  |  |  | cfDNA*:ti,ab,kw |
|  |  |  | ‘Cell free DNA’:ti,ab,kw |
|  |  |  | ‘Circulating cell free tumor DNA’:ti,ab,kw |
|  |  |  | ‘Circulating cell free tumour DNA’:ti,ab,kw |
|  |  |  | ccfDNA:ti,ab,kw |
|  |  |  | ‘Liquid biopsy’/exp |
|  |  |  | ‘Liquid biops*’:ti,ab,kw |
|  |  |  | ‘Cell free nucleic acid*’:ti,ab,kw |
