# Supplementary material for: Early diagnosis of ovarian cancer based on methylation profiles in peripheral blood cell-free DNA: a systematic review
Source: Clin Epigenetics. 2023 Feb 14;15:24. doi: 10.1186/s13148-023-01440-w (PMC9926627; doi:10.1186/s13148-023-01440-w)
Supplement: Supplementary file 2 — Additional file 2: Table S2: Quality assessment of included studies using the Newcastle-Ottawa Scale. [file 13148_2023_1440_MOESM2_ESM.docx]

# **Additional file 2**

**Table Additional file 2**: Quality assessment of included studies using the Newcastle-Ottawa Scale. The scale was modified to only include point two under exposure and the total score was corrected for this. Good quality assessment was defined as 3-4 stars in *Selection*, 1-2 stars in *Comparability*, and 1 star in *Exposure*. Moderate quality assessment was defined as 2 stars in *Selection*, 1-2 stars in *Comparability*, and 0-1 star in *Exposure*. Poor quality assessment was defined as 0-1 star in *Selection*, 0 stars in *Comparability*, and 0-1 star in *Exposure.*

| Author  (*et al.*) | Selection | | | | Comparability | Exposure | Total score | Quality assessment |
| --- | --- | --- | --- | --- | --- | --- | --- | --- |
|  | Definition  Cases | Representativeness Cases | Selection Controls | Definition Controls | Comparability of Cases and Controls  1) Age  2) Sample volume | Same method for cases and controls |  |  |
| de Caceres (65) | * | - | - | * | ** | * | 5/7 | Moderate |
| Su (18) | * | - | * | * | - * | * | 5/7 | Moderate |
| Melnikov (33) | * | - | * | * | ** | * | 6/7 | Good |
| BonDurant (43) | * | - | - | * | - * | * | 4/7 | Moderate |
| Campan (66) | - | - | - | * | -* | - | 2/7 | Poor |
| Häfner (67) | * | - | - | * | -* | * | 4/7 | Moderate |
| Liggett (26) | * | - | - | * | ** | * | 5/7 | Moderate |
| Dong (68) | * | * | - | * | -* | * | 5/7 | Good |
| Wang (69) | * | - | - | - | -- | * | 2/7 | Poor |
| Zhang (70) | * | - | - | * | -* | * | 4/7 | Moderate |
| Wu (49) | * | - | * | * | -- | * | 4/7 | Moderate-Poor |
| Zhou (34) | * | - | - | * | ** | * | 5/7 | Moderate |
| Wang (19) | * | - | - | * | -- | * | 3/7 | Moderate-Poor |
| Giannopoulou (21) | * | - | - | * | -* | * | 4/7 | Moderate |
| Swellam (45) | * | - | * | * | ** | * | 6/7 | Good |
| Wang (20) | * | - | * | * | -* | * | 5/7 | Good |
| Widschwendter (29) | * | * | - | * | -- | * | 4/7 | Moderate-Poor |
| Giannopoulou (22) | * | - | - | * | -* | * | 4/7 | Moderate |
| Dvorská (71) | * | - | - | * | *- | * | 4/7 | Moderate |
| Kumar (42) | * | - | - | * | ** | * | 5/7 | Moderate |
| Liu (11) | * | * | * | * | ** | * | 7/7 | Good |
| Miller (47) | * | - | - | * | *- | * | 4/7 | Moderate |
| Singh (27) | * | - | - | * | ** | * | 5/7 | Moderate |
| Faaborg (28) | * | - | - | * | -* | * | 4/7 | Moderate |
| Miller (48) | * | - | - | * | -* | * | 4/7 | Moderate |
| Singh (46) | * | * | - | * | -* | * | 5/7 | Good |
| Tserpeli (44) | * | - | - | * | -* | * | 4/7 | Moderate |
| Marinelli (39) | * | - | - | * | ** | * | 5/7 | Moderate |
| Tomeva (30) | * | - | - | * | -* | * | 4/7 | Moderate |
