# Supplementary material for: Early diagnosis of ovarian cancer based on methylation profiles in peripheral blood cell-free DNA: a systematic review
Source: Clin Epigenetics. 2023 Feb 14;15:24. doi: 10.1186/s13148-023-01440-w (PMC9926627; doi:10.1186/s13148-023-01440-w)
Supplement: Supplementary file 3 — Additional file 3: Table S3: Gene-specific methylation biomarkers investigated in plasma or serum from OC and controls reported in only one study. [file 13148_2023_1440_MOESM3_ESM.docx]

# **Additional file 3**

**Table Additional file 3:** Gene-specific methylation biomarkers investigated in plasma or serum from OC and controls, including methods, methylation frequency, and performance for early diagnosis of OC reported in only one study. 95 % exact confidence intervals (CI) have been calculated where data were available.

| Gene | Author  (*et al.*) | Method | Case/control | Meth OC | Meth control | Sensitivity %  (95% CI) | Specificity %  (95% CI) |
| --- | --- | --- | --- | --- | --- | --- | --- |
| *ALKBH3* | Tomeva (30) | Real-Time qPCR | 19/8^h^ (T) | N/A | N/A | N/A | N/A |
| *AGRN* | Marinelli (39) | TELQAS | 91/91^h^ (T) | N/A | N/A | N/A | N/A |
| *APKinase* | de Caceres (65) | MSP | 50/10^b^/20^h^ (T) | N/A | N/A | N/A | N/A |
| *BCAT1* | Marinelli (39) | TELQAS | 91/91^h^ (T) | N/A | N/A | N/A | N/A |
| *CALCA* | Liggett (26) | MSRE/PCR/microarray | 30/30^b^/30^h^ (T) | N/A | N/A | N/A | N/A |
| *CAPN2* | Marinelli (39) | TELQAS | 91/91^h^ (T) | N/A | N/A | N/A | N/A |
| *CDKN1C* | Liggett (26) | MSRE/PCR/microarray | 30/30^b^/30^h^ (T) | N/A | N/A | N/A | N/A |
| *CDO1* | Marinelli (39) | TELQAS | 91/91^h^ (T) | N/A | N/A | N/A | N/A |
| *CELF2* | Marinelli (39) | TELQAS | 91/91^h^ (T) | N/A | N/A | N/A | N/A |
| *COL23A1* | Widschwendter (29) | Targeted NGS | 29/119^b^/21^h^ (T)  48/154^b^/41^h^ (V) | N/A | N/A | N/A | N/A |
| *C2CD4D* | Widschwendter (29) | Targeted NGS | 29/119^b^/21^h^ (T)  48/154^b^/41^h^ (V) | N/A | N/A | N/A | N/A |
| *DKN1C* | Liggett (26) | MSRE/PCR/microarray | 30/30^b^/30^h^ (T) | N/A | N/A | N/A | N/A |
| *DLEC1* | Swellam (45) | MSP | 90/50^b^/30^h^ (T) | 86/90 | 20/50^b^  0/30^h^ | 95.6  (89.0-98.8) | 75.0  (64.1-84.0) |
| *EP300* | Liggett (26) | MSRE/PCR/microarray | 30/30^b^/30^h^ (T) | N/A | N/A | N/A | N/A |
| *ESR1* | Giannopoulou (22) | Real-time MSP | 50/51^h^ ^‡^ (T) | 19/50 | 1/51 | 38.0*  (24.7-52.8) | 98.0*  (89.6-99.9) |
| *FAIM2* | Marinelli (39) | TELQAS | 91/91^h^ (T) | N/A | N/A | N/A | N/A |
| *GATA5* | Tomeva (30) | Real-Time qPCR | 19/8^h^ (T) | N/A | N/A | N/A | N/A |
| *GPRIN1* | Marinelli (39) | TELQAS | 91/91^h^ (T) | N/A | N/A | N/A | N/A |
| *GSTP1* | Tomeva (30) | Real-Time qPCR | 19/8^h^ (T) | N/A | N/A | N/A | N/A |
| *GYPC* | Marinelli (39) | TELQAS | 91/91^h^ (T) | N/A | N/A | N/A | N/A |
| *IFFO1* | Campan (66) | Digital MethyLight | 16/8^h^ (T) | 16/16 | 2/8 | 100*  (79.4-100) | 75.0*  (34.9-96.8) |
| *LMX1A* | Su (18) | MSP | 26/20^b^ (T) | 8/26 | 2/20^b^ | 30.8*  (14.3-51.8) | 90.0*  (68.3-98.8) |
| *MDR1* | Tomeva (30) | Real-Time qPCR | 19/8^h^ (T) | N/A | N/A | N/A | N/A |
| *MGMT* | Tomeva (30) | Real-Time qPCR | 19/8^h^ (T) | N/A | N/A | N/A | N/A |
| *MLH1* | Tomeva (30) | Real-Time qPCR | 19/8^h^ (T) | N/A | N/A | N/A | N/A |
| *PAX5* | Melnikov (33) | MSRE/PCR/microarray | 33/33^h^ (T) | 26/33* | 19/33* | 78.8*  (61.1-91.0) | 42.4*  (25.5-60.8) |
| *PTEN* | Dvorská (71) | Pyrosequencing | 33/5^b^/9^h^ (T) | N/A | N/A | N/A | N/A |
| *p14ARF* | de Caceres (65) | MSP | 50/10^b^/20^h^ (T) | N/A | N/A | N/A | N/A |
| *p16INK4a* | de Caceres (65) | MSP | 50/10^b^/20^h^ (T) | N/A | N/A | N/A | N/A |
| *RASSF1* | Dvorská (71) | Pyrosequencing | 33/5^b^/9^h^ (T) | N/A | N/A | N/A | N/A |
| *RASSF2A* | Wu (49) | MSP | 47/14^b^ (T) | 14/47 | 0/14 | 29.8*  (17.3-44.9) | 100*  (76.8-100) |
| *RIPPLY3* | Marinelli (39) | TELQAS | 91/91^h^ (T) | N/A | N/A | N/A | N/A |
| *SEPT9* | Tomeva (30) | Real-Time qPCR | 19/8^h^ (T) | N/A | N/A | N/A | N/A |
| *SFRP1* | Su (18) | MSP | 26/20^b^ (T) | 8/26 | 2/20^b^ | 30.8*  (14.3-51.8) | 90.0*  (68.3-98.8) |
| *SFRP2* | Su (18) | MSP | 26/20^b^ (T) | 4/26 | 2/20^b^ | 15.4*  (4.36-34.9) | 90.0*  (68.3-98.8) |
| *SFN* | Tomeva (30) | Real-Time qPCR | 19/8^h^ (T) | N/A | N/A | N/A | N/A |
| *SHOX2* | Tomeva (30) | Real-Time qPCR | 19/8^h^ (T) | N/A | N/A | N/A | N/A |
| *SIM2* | Marinelli (39) | TELQAS | 91/91^h^ (T) | N/A | N/A | N/A | N/A |
| *SLFN11* | Tserpeli (44) | Real-time MSP | 84/49/27^h^ (T) | 3/80  7/48 | 0/27 | 3.80  (0.78-10.6)  14.6  (6.07-27.8) | 100  (87.2-100) |
| *SLIT2* | Dong (68) | MSP | 36/25^h^ (T) | 27/36 | 0/25 | 75.0*  (57.8-87.9) | 100*  (86.3-100) |
| *SRC* | Marinelli (39) | TELQAS | 91/91^h^ (T) | N/A | N/A | N/A | N/A |
| *THBS1* | Melnikov (33) | MSRE/PCR/microarray | 33/33^h^ (T) | 30/33* | 17/33* | 90.9*  (75.7-98.1) | 48.5*  (30.8-66.5) |
| *USP44* | Tserpeli (44) | Real-time qMSP | 84/49/27^h^ (T) | 6/80  8/49 | 0/27 | 7.50  (2.80-15.6)  16.3  (7.32-29.7) | 100  (87.2-100) |
| *VIM* | Tomeva (30) | Real-Time qPCR | 19/8^h^ (T) | N/A | N/A | N/A | N/A |
| *WNT6* | Widschwendter (29) | Targeted NGS | 29/119^b^/21^h^ (T)  48/154^b^/41^h^ (V) | N/A | N/A | N/A | N/A |

Abbreviations: b: benign, bl: Borderline tumor. h: healthy. Meth: methylated. MSP: Methylation-specific-PCR. MSRE: Methylation-sensitive restriction enzyme. MS-HRMA: methylation-sensitive high resolution melting analysis. N/A: Not available. PCR: Polymerase Chain Reaction. qMSP: Quantitative Methylation-specific PCR. qPCR: Quantitative PCR. TELQAS: Target Enrichment Long-probe Quantitative Amplified Signal. T: Training/test cohort. V: Validation cohort.
* Extrapolated calculations based on available data.
‡ Appear to be reuse of cohort/data from Giannopoulou *et al.* (21).
